# Urbanization, Altitude and Cardiovascular Risk

**DOI:** 10.5334/gh.1130

**Published:** 2022-06-21

**Authors:** Antonio Bernabe-Ortiz, Rodrigo M. Carrillo-Larco

**Affiliations:** 1 CRONICAS Center of Excellence in Chronic Diseases, Universidad Peruana Cayetano Heredia, Lima, Peru; 2 Universidad Científica del Sur, Lima, Peru; 3 Department of Epidemiology and Biostatistics, School of Public Health, Imperial College London, London, UK; 4 Universidad Continental, Lima, Peru

**Keywords:** Cardiovascular disease, cardiovascular risk, altitude, urbanization, Peru

## Abstract

**Background:**

There is limited information regarding the variation of the cardiovascular (CV) risk, that combines multiple risk factors in one metric, according to urbanization and altitude.

**Objective:**

To assess and disentangle the potential association between urbanization and altitude with absolute CV risk using Peruvian nationally representative surveys.

**Methods:**

Pooled analysis of Peruvian Demographic Health Surveys (from 2014 to 2020), including subjects aged between 40 and 74 years, was conducted. The outcome of interest was the 10-year predicted absolute CV risk based on the non-laboratory version of the World Health Organization (WHO) and split into <10% and ≥10%. The exposures were urbanization (rural or urban) and altitude (<500 meters above the sea level [m.a.s.l.], between 500 and 2,499 m.a.s.l, between 2,500 and 3,499 m.a.s.l., and ≥3,500 m.a.s.l.). Crude and adjusted Poisson regression models were built to assess the associations of interest, reporting prevalence ratios (PR) and 95% confidence intervals (95% CI).

**Results:**

Data of 80,409 subjects, mean age 54.3 (SD: 8.8) and 42,640 (54.4%) females were analyzed. Regarding urbanization, 30,722 (25.4%) subjects were from rural areas, and 60.6% lives at <500 m.a.s.l., whereas only 9.3% lives at ≥3500 m.a.s.l. The 10-year predicted absolute CV risk mean was 4.5% (SD: 3.1), and 7.8% had a CV risk ≥10%. In multivariable model, urbanization, mainly rurality (PR = 0.89; 95%CI: 0.81–0.97) and altitude (PR = 0.82; 95%CI: 0.75–0.90 for those living between 2,500–3,490 m.a.s.l. and PR = 0.68; 95%CI: 0.60–0.76 for those living ≥3,500 m.a.s.l) were factors independently associated with CV risk. Urbanization was an effect modifier of the association between altitude and CV risk with a greater effect in urban settings.

**Conclusion:**

Urbanization, specifically rurality, and high-altitude, mainly ≥2,500 m.a.s.l., were factors independently associated with lower predicted CV risk.

## Introduction

Globally, the burden of cardiovascular disease (CVD), including angina pectoris, myocardial infarction, heart failure, and stroke, is a public health concern. In high income countries, the age-standardized CVD death rate has decreased during the last three decades, whereas it has not changed in low- and middle-income countries during the same period [1]. However, over three quarters of heart disease and stroke-related deaths occur in resource-constrained settings [2].

The distribution of main risk factors for CVD (i.e., hypertension, type 2 diabetes, hypercholesterolemia, obesity, and smoking) vary widely between global regions, countries and even within a country [3]. Such differences have been imputed to geographical variation, specifically, to urbanization, i.e., whether an individual lives in a rural or urban setting [4], and, altitude, i.e., how far above sea level the subject resides [5]. Thus, for example, in Peru, there is a clear heterogeneity in the progression towards hypertension depending on urbanization and altitude [6], whereas abdominal obesity prevalence seems to decrease with altitude [7], but high altitude has been associated with greater type 2 diabetes incidence [8]. On the other hand, urbanization, mainly rurality, and not altitude has been associated with dyslipidemia traits [9].

Previous studies have assessed the role that altitude plays on CVD risk. A study conducted in the US reported that living at higher altitude (over 1500 meters above the sea level [m.a.s.l.]) had a protective effect on ischemic heart disease [10]. Similarly, an ecological study in Austria reported that moderate altitude (1000–2000 m.a.s.l.) elicited beneficial effect on all cause-mortality, mainly from circulatory diseases and cancer [11]. In a small study, high altitude was negatively associated with Framingham 10-year risk score [12]. Nevertheless, there is limited information regarding the potential variation of the absolute cardiovascular (CV) risk, that combines multiple risk factors in one metric and guides primary prevention of CVD, according to altitude, but also including urbanization (i.e., rural vs. urban settings).

For purposes of establishing health priorities, especially in resource-constrained settings, it is important to understand the local epidemiology of CVD risk including within-country heterogeneity of noncommunicable conditions profiles and their factors [13]. This information may provide evidence to establish suitable interventions along with appropriate goals and targets [14,15]. Peru is an ideal country to examine the interaction of altitude and residence with CVD risk. As a result, this manuscript aimed to evaluate the potential association between urbanization and altitude with absolute CV risk using nationally representative surveys conducted in Peru. In addition, we assess whether altitude is an effect modifier of the association between altitude and CVD risk. We hypothesized that CVD risk is negatively associated with altitude and rural residence, and the association between altitude and CVD risk differs by urbanization.

## Methods

### Study design

Information from the Peruvian Demographic Health Survey (DHS or ENDES in Spanish) was used. The survey is annually conducted by the National Institute of Statistics and Informatics (INEI in Spanish), and collects information on variables including poverty, fertility, violence, and health. For this analysis, data from the DHS 2014 to 2020 were pooled and analyzed.

### Study population and selection criteria

The Peruvian DHS utilized a bietapic sampling approach. In rural areas, the primary sampling units comprise clusters of 500 to 2,000 subjects, whereas the secondary sampling units are households within each of these clusters. However, in urban areas, the approach is a little different: the primary sampling units are blocks or groups of blocks with more than 2,000 subjects within an average of 140 households, whilst secondary sampling units comprise households as in rural areas [16].

The sampling process and techniques used are described in the technical documents of the DHS [17]. The Peruvian DHS is conducted every year; however, the sample frame used for participants’ selection is updated every three years (i.e., 2012–2014, 2015–2017, 2018–2020). The method used for sampling is known as the method of the cube, which allow obtaining samples with estimates of totals approximately equal to the characteristics of the population of the survey that replicates the population structure within the selected sample considering the groups of age, sex and other balance variables.

The present analysis included subjects aged between 40 and 74 years as prediction equations have been validated in this age range [18], with complete information to estimate the 10-year cardiovascular risk (i.e., age, sex, current smoking status, and body mass index), as well as urbanization and altitude information.

## Definition of variables

### Outcome

The outcome of interest was the 10-year predicted absolute CV risk. Since the Peruvian DHS did not collect blood samples for fasting glucose or total cholesterol, we decided to utilize the non-laboratory version of the World Health Organization (WHO) 10-year risk of cardiovascular disease (CVD) [19]. Such risk score used data from the Emerging Risk Factors Collaboration and recalibrates the predicted risk scores to reflect the expected 10-year risk in contemporary population in 21 global regions, including sex, age (in years), smoking status and body mass index (kg/m^2^). CV risk was estimated utilizing the *whocvdrisk* command in STATA. For analysis purposes, the CV risk score was described according to standard thresholds (<5%, between 5% to <10%, between 10% to <20%, and ≥20%), and then categorized using a cutoff of 10% as usually individuals with such CV risk level or above should receive an evaluation using laboratory-based charts [18].

#### Exposure

Two were the exposures in our analysis. The first one was urbanization, with two potential categories, rural or urban, as defined by INEI criteria. Accordingly, a rural setting is the area with no more than 100 households contiguously grouped and is not a district capital, or an area having ≥100 households, but these are dispersed or scattered without forming blocks [17]; whereas the remainder settings were considered urban areas for analyses purposes.

The second exposure was altitude above the sea level, which was collected using a Global Positioning System (GPS) utilized a standardized procedure [20]. Briefly, GPS data (i.e., longitude, latitude, and altitude) are collected in the household of each participant at one meter apart from the main door and using the data of at least five satellites to guarantee appropriate quality. For analyses purposes, and because the emphasis was done in high-altitude settings, this variable was split into four categories: <500 meters above the sea level (m.a.s.l), between 500 and 2,499 m.a.s.l, between 2,500 and 3,499 m.a.s.l., and ≥3,500 m.a.s.l.

#### Co-variables

Other variables were used in the analyses as potential confounders. Such variables were: sex (female vs. male); age (40–49, 50–59, 60–69, and 70+ years); education (<7 years, 7–11 years, ad 12+ years); wealth index, as a proxy of socioeconomic position, built on household assets and services available as reported by participants. The wealth index was built for each year and categorized into quintiles using the approach reported by Rutstein and Johnson [21]; and survey year.

In addition, for descriptive purposes, we also included current smoking status, defined as the reporting of the consumption of at least one cigarette in the last 12 months (no vs. yes); alcohol use, based on the self-report of the consumption of any alcoholic beverage (beer or equivalent) during the last 30 days (no vs. yes), and hypertension status, defined accordingly to the JNC-7 (i.e., systolic blood pressure ≥140 mm Hg, or diastolic blood pressure ≥90 mm Hg, or previous hypertension diagnosis done by a physician) [22].

### Procedures

All the questionnaires and tools were applied by trained staff using tablets (LENOVO B600F or B600H) using a special software developed by INEI [20]. In addition, GPS data collection was also available with the software used.

Anthropometrical procedures (height, weight and blood pressure) were collected by trained personnel [23]. Height was measured using a standard stadiometer, whereas weight was measured utilizing an electronic scale (SECA model 872, 874 and 878). Blood pressure levels were collected using a digital monitor (OMRON, model HEM-713). Two types of cuffs were utilized depending on the arm circumference of participants. Blood pressure measurements were assessed twice with the participant sitting and their right arm resting on a flat surface at the heart level. The first measurement was taken after a resting period of at least five minutes, and the second measurement was done two minutes after the first measurement [23].

### Statistical analysis

Statistical analyses were conducted using STATA 16 for Windows (StataCorp, College Station, TX, USA). All the analyses took into account the multistage complex design of the sample, and subpopulation (*subpop* command) analyses were also pursued [24].

Initially, a description of the study population, including a comparison between those included and not included in the analyses, was conducted. Means and standard deviation (SD) and absolute and relative frequencies were utilized to describe numerical and categorical variables, respectively. In addition, the prevalence of variables of interest were reported as well as their respective 95% confidence intervals (95% CI).

Then, the description of the study population by the exposures (i.e., urbanization and altitude) and the outcome (i.e., absolute CV risk) was also conducted. Comparisons were carried out utilizing the Chi-squared test corrected for the survey design with the second-order correction of Rao and Scott [25].

To assess the associations of interest, crude and adjusted Poisson regression models were built. Multivariable models were adjusted by age, sex, education, wealth index, and survey year. In addition, urbanization and altitude were evaluated in the same regression model to examine if both variables were independently associated with CV risk. Finally, urbanization was evaluated as potential effect modifier of the association between altitude and CVD risk using the *testparm* command in STATA. Prevalence ratios and 95% CI were reported. A p-value <0.05 was considered statistically significant.

## Results

### Characteristics of the study population

Data of 86,206 participants fulfilled the inclusion criteria and were pooled using information from ENDES 2014 to 2020. Of them, 143 records were excluded because pregnancy and 5,654 records because of lack of data of interest (i.e., there was not information about altitude, urbanization or variables associated with estimation of the CV risk). Comparison of study characteristics between individuals included and excluded in the analyses are available in Online Supplement E-Table 1. As a result, the information of 80,409 subjects, mean age 54.3 (SD: 8.8), 42,640 (54.4%) females, and 18,098 (29.1%) with 12+ years of education, were further analyzed.

### Urbanization and altitude

A total of 30,722 (25.4%; 95% CI: 24.7% – 26.2%) individuals were from rural areas. Age (p < 0.001), sex (p < 0.001), education (p < 0.001), wealth index (p < 0.001), alcohol use (p < 0.001), hypertension (p < 0.001), and survey year (p < 0.001) were associated with urbanization in bivariable analyses (Table 1).

**Table 1:** Description of the study population by urbanization: Comparison taking into account multi-stage design.


	URBAN	RURAL	P-VALUE

	(N = 49,687)	(N = 30,722)	

**Age**			<0.001

40 – 49 years	22,001 (38.8%)	11,630 (34.5%)	

50 – 59 years	14,326 (30.2%)	9,104 (30.6%)	

60 – 69 years	10,072 (23.0%)	7,119 (24.8%)	

70+ years	3,288 (8.0%)	2,869 (10.1%)	

**Sex**			<0.001

Male	22,781 (44.7%)	14,988 (48.0%)	

Female	26,906 (55.3%)	15,734 (52.0%)	

**Education**			<0.001

<7 years	13,109 (24.5%)	18,211 (70.5%)	

7 – 11 years	18,742 (39.7%)	6,492 (22.7%)	

12+ years	16,166 (35.8%)	1,932 (6.8%)	

**Wealth index**			<0.001

First quintile	3,086 (4.2%)	14,731 (49.4%)	

Second quintile	4,418 (6.8%)	11,499 (36.0%)	

Third quintile	10,599 (19.1%)	2,952 (9.2%)	

Fourth quintile	14,512 (31.9%)	1,074 (3.7%)	

Fifth quintile	17,072 (38.0%)	466 (1.7%)	

**Current smoking**			0.72

No	42,035 (85.3%)	25,720 (85.5%)	

Yes	7,652 (14.7%)	5,002 (14.5%)	

**Alcohol use**			<0.001

No	32,500 (65.1%)	23,092 (75.2%)	

Yes	17,159 (34.9%)	7,609 (24.8%)	

**Hypertension**			<0.001

No	34,536 (66.3%)	23,237 (74.7%)	

Yes	15,151 (33.7%)	7,485 (25.3%)	

**Survey year**			<0.001

2014	7,043 (11.8%)	4,867 (14.5%)	

2015	7,256 (14.8%)	4,170 (15.7%)	

2016	7,252 (14.2%)	4,226 (15.1%)	

2017	7,366 (15.7%)	4,511 (14.7%)	

2018	7,864 (16.6%)	4,681 (15.2%)	

2019	7,518 (16.0%)	4,877 (14.9%)	

2020	5,388 (10.9%)	3,390 (9.9%)	


Regarding altitude, 60.6% (95% CI: 59.4% – 61.8%) of subjects were living at <500 m.a.s.l., whereas only 9.3% (95% CI: 8.7% – 10.0%) were living at ≥3500 m.a.s.l. All the variables, except survey year, were associated with altitude in bivariable model (Table 2).

**Table 2:** Description of the study population by altitude: Comparison taking into account multi-stage design.


	ALTITUDE (METERS ABOVE SEA LEVEL)				P-VALUE

	<500	500 – 2499	2500 – 3499	≥3500	

	(N = 38,188)	(N = 16,198)	(N = 15,301)	(N = 10,722)	

**Age**					<0.001

40 – 49 years	16,338 (38.3%)	7,123 (38.8%)	6,245 (36.9%)	3,925 (34.0%)	

50 – 59 years	11,362 (30.2%)	4,645 (30.4%)	4,316 (30.5%)	3,107 (30.5%)	

60 – 69 years	8,001 (23.5%)	3,261 (22.4%)	3,335 (22.9%)	2,594 (25.2%)	

70+ years	2,487 (8.0%)	1,169 (8.4%)	1,405 (9.7%)	1,096 (10.3%)	

**Sex**					<0.001

Male	18,266 (46.0%)	7,844 (47.2%)	6,870 (43.3%)	4,789 (43.7%)	

Female	19,922 (54.0%)	8,354 (52.8%)	8,431 (56.7%)	5,933 (56.3%)	

**Education**					<0.001

<7 years	11,552 (25.8%)	6,719 (44.4%)	7,461 (52.4%)	5,588 (60.0%)	

7 – 11 years	14,886 (40.6%)	4,630 (30.5%)	3,361 (25.3%)	2,357 (25.9%)	

12+ years	10,464 (33.6%)	3,764 (25.1%)	2,643 (22.3%)	1,227 (14.1%)	

**Wealth index**					<0.001

First quartile	4,273 (6.3%)	3,684 (22.5%)	5,445 (31.1%)	4,415 (41.0%)	

Second quartile	4,496 (7.5%)	3,161 (19.1%)	4,552 (26.3%)	3,708 (30.7%)	

Third quartile	7,489 (17.6%)	2,804 (16.8%)	2,075 (14.9%)	1,183 (12.6%)	

Fourth quartile	10,069 (30.9%)	3,067 (20.0%)	1,720 (14.9%)	730 (8.0%)	

Fifth quartile	11,861 (37.7%)	3,482 (21.6%)	1,509 (12.8%)	686 (7.7%)	

**Current smoking**					<0.001

No	31,748 (84.8%)	13,827 (85.5%)	13,138 (87.0%)	9,042 (86.4%)	

Yes	6,440 (15.2%)	2,371 (14.5%)	2,163 (13.0%)	1,680 (13.6%)	

**Alcohol use**					<0.001

No	24,903 (64.5%)	11,433 (70.6%)	10,917 (71.4%)	8,339 (77.2%)	

Yes	13,259 (35.5%)	4,756 (29.4%)	4,379 (28.6%)	2,374 (22.8%)	

**Hypertension**					<0.001

No	26,208 (65.4%)	11,664 (69.9%)	11,582 (74.4%)	8,319 (76.4%)	

Yes	11,980 (34.6%)	4,534 (30.1%)	3,719 (25.6%)	2,403 (23.6%)	

**Survey year**					0.21

2014	5,547 (12.1%)	2,204 (11.9%)	2,301 (13.4%)	1,858 (14.8%)	

2015	5,616 (14.8%)	2,345 (15.5%)	2,069 (15.0%)	1,396 (15.7%)	

2016	5,513 (14.2%)	2,455 (15.4%)	2,118 (14.6%)	1,392 (14.6%)	

2017	5,610 (15.5%)	2,501 (15.9%)	2,244 (14.6%)	1,522 (15.1%)	

2018	5,972 (16.4%)	2,476 (15.8%)	2,404 (16.2%)	1,693 (15.9%)	

2019	5,751 (16.1%)	2,421 (15.0%)	2,506 (16.0%)	1,717 (14.5%)	

2020	4,179 (10.9%)	1,796 (10.5%)	1,659 (10.2%)	1,144 (9.4%)	


### Absolute cardiovascular risk

The average 10-year predicted absolute CV risk was 4.5% (SD: 3.1), with values ranging from 0.5% to 37.4%. In addition, 56,333 (66.5%; 95% CI: 66.0% – 67.1%) had a cardiovascular risk <5%; 18,885 (25.7%; 95% CI: 25.1% – 26.2%) had a risk between 5% and <10%; 5,012 (7.5%; 95% CI: 7.2% – 7.9%) had a risk between 10% and <20%; and 179 (0.3%; 95% CI: 0.2% – 0.4%) had a risk ≥20%. Age (p < 0.001), sex (p < 0.001), education (p < 0.001), wealth index (p < 0.001), current smoking (p < 0.001), and hypertension (p < 0.001) were factors associated with an absolute CV risk ≥10% in bivariable analysis (Table 3).

**Table 3:** Description of the study population by cardiovascular risk: Comparison taking into account multi-stage design.


	10-YEAR CARDIOVASCULAR RISK		P-VALUE

	LOW (<10%)	HIGH (≥10%)	

	(N = 75,218)	(N = 5,191)	

**Age**			<0.001

40 – 49 years	33,609 (54.5%)	22 (4.2%)	

50 – 59 years	23,147 (36.4%)	283 (18.2%)	

60 – 69 years	15,240 (9.0%)	1,951 (52.0%)	

70+ years	3,222 (0.1%)	2,935 (25.5%)	

**Sex**			<0.001

Male	33,902 (43.3%)	3,867 (72.7%)	

Female	41,316 (56.7%)	1,324 (27.3%)	

**Education**			<0.001

<7 years	28,885 (34.5%)	2,435 (43.6%)	

7 – 11 years	23,973 (36.2%)	1,261 (30.5%)	

12+ years	17,200 (29.3%)	898 (25.9%)	

**Wealth index**			<0.001

First quintile	16,668 (15.8%)	1,149 (14.0%)	

Second quintile	14,945 (14.4%)	972 (12.4%)	

Third quintile	12,794 (16.7%)	757 (15.5%)	

Fourth quintile	14,554 (24.6%)	1,032 (25.9%)	

Fifth quintile	16,257 (28.5%)	1,281 (32.2%)	

**Current smoking**			<0.001

No	64,198 (86.5%)	3,557 (71.8%)	

Yes	11,020 (13.5%)	1,634 (28.2%)	

**Alcohol use**			0.20

No	51,977 (67.5%)	3,615 (69.0%)	

Yes	23,198 (32.5%)	1,570 (31.0%)	

**Hypertension**			<0.001

No	56,686 (72.7%)	1,087 (18.6%)	

Yes	18,532 (27.3%)	4,104 (81.4%)	

**Survey year**			0.07

2014	10,981 (12.4%)	929 (13.0%)	

2015	10,780 (15.2%)	646 (12.9%)	

2016	10,790 (14.5%)	688 (14.1%)	

2017	11,057 (15.3%)	820 (16.8%)	

2018	11,773 (16.2%)	772 (16.6%)	

2019	11,616 (15.7%)	779 (16.2%)	

2020	8,221 (10.7%)	557 (10.4%)	


### Urbanization, altitude and absolute cardiovascular risk

After controlling for age, sex, education, wealth index, and survey year, higher altitude was associated with lower absolute cardiovascular risk (Table 4). Thus, the proportion of subjects with CV risk ≥10% was lower among those living between 2,500 – 3,490 m.a.s.l. (PR = 0.81; 95% CI: 0.74 – 0.88) compared to those living <500 m.a.s.l., and such proportion was much lower among those living at ≥3,500 m.a.s.l (PR = 0.66; 95% CI: 0.59 – 0.73). Additionally, in multivariable model, the proportion of subjects with CV risk ≥10% living in rural area was lower (PR = 0.82; 95% CI: 0.75 – 0.89) compared to those living in urban areas. When urbanization and altitude were included in the same regression model, both variables were independently associated with CV risk (Table 4).

**Table 4:** Association between urbanization, altitude and cardiovascular risk: Crude and adjusted models. Models were built taking into account the multi-stage design of the surveys.


	10-YEAR CARDIOVASCULAR RISK			

	LOW (<10%)	HIGH (≥10%)	CRUDE MODEL	ADJUSTED MODEL*

	(N = 66,997)	(N = 4,634)	PR (95% CI)	PR (95% CI)

**Urbanization and altitude in different models**				

***Urbanization***				

Urban	46,386 (91.8%)	3,301 (8.2%)	1 (Reference)	1 (Reference)

Rural	28,832 (93.4%)	1,890 (6.6%)	**0.80 (0.74 – 0.86)**	**0.82 (0.75 – 0.89)**

***Altitude***				

<500 m.a.s.l.	35,546 (91.5%)	2,642 (8.5%)	1 (Reference)	1 (Reference)

500 – 2,499 m.a.s.l.	15,170 (92.5%)	1,028 (7.5%)	**0.87 (0.79 – 0.97)**	0.95 (0.87 – 1.03)

2,500 – 3,499 m.a.s.l.	14,382 (93.5%)	919 (6.5%)	**0.77 (0.69 – 0.85)**	**0.81 (0.74 – 0.88)**

≥3,500 m.a.s.l.	10,120 (94.4 %)	602 (5.6%)	**0.66 (0.59 – 0.74)**	**0.66 (0.59 – 0.73)**

**Urbanization and altitude in the same model**				

***Urbanization***				

Urban			1 (Reference)	1 (Reference)

Rural			**0.91 (0.84 – 0.99)**	**0.89 (0.81 – 0.97)**

***Altitude***				

<500 m.a.s.l.			1 (Reference)	1 (Reference)

500 – 2,499 m.a.s.l.			**0.90 (0.81 – 0.99)**	0.96 (0.89 – 1.05)

2,500 – 3,499 m.a.s.l.			**0.80 (0.72 – 0.88)**	**0.82 (0.75 – 0.90)**

≥3,500 m.a.s.l.			**0.69 (0.62 – 0.78)**	**0.68 (0.60 – 0.76)**


* Adjusted by age, sex, education, wealth index, and survey year.

Urbanization was an effect modifier of the association between altitude and CV risk (p < 0.001). Thus, the protective effect of altitude on CV risk was seen ≥2500 m.a.s.l among those living in urban areas, whereas such association was observable only among those living ≥3500 m.a.s.l. in rural settings (See Table 5).

**Table 5:** Association between altitude and cardiovascular risk by urbanization: Crude and adjusted models. Models were built taking into account the multi-stage design of the surveys.


	10-YEAR CARDIOVASCULAR RISK			

	LOW (<10%)	HIGH (≥10%)	CRUDE MODEL	ADJUSTED MODEL*

	(N = 66,997)	(N = 4,634)	PR (95% CI)	PR (95% CI)

**Urban area**				

***Altitude***				

<500 m.a.s.l.	29,704 (91.3%)	2,293 (8.7%)	1 (Reference)	1 (Reference)

500 – 2,499 m.a.s.l.	8,469 (92.5%)	558 (7.5%)	**0.86 (0.75 – 0.97)**	0.93 (0.84 – 1.03)

2,500 – 3,499 m.a.s.l.	5,525 (93.4%)	321 (6.6%)	**0.75 (0.65 – 0.86)**	**0.76 (0.67 – 0.86)**

≥3,500 m.a.s.l.	2,688 (95.4%)	129 (4.6%)	**0.53 (0.41 – 0.67)**	**0.69 (0.56 – 0.86)**

**Rural area**				

***Altitude***				

<500 m.a.s.l.	5,842 (93.8%)	349 (6.2%)	1 (Reference)	1 (Reference)

500 – 2,499 m.a.s.l.	6,701 (92.6%)	470 (7.4%)	**1.20 (1.01 – 1.44)**	1.16 (0.99 – 1.33)

2,500 – 3,499 m.a.s.l.	8,857 (93.5%)	598 (6.5%)	1.06 (0.89 – 1.25)	1.02 (0.88 – 1.17)

≥3,500 m.a.s.l.	7,432 (93.9%)	473 (6.1%)	0.99 (0.83 – 1.18)	**0.76 (0.65 – 0.89)**


* Adjusted by age, sex, education, wealth index, and survey year.

## Discussion

### Main results

Our study reports evidence that both, urbanization, specifically rurality, and altitude, mainly settings at ≥2,500 m.a.s.l., had a lower proportion of individuals having a CV risk ≥10% in multivariable models. Moreover, the association of urbanization with CV risk was independent of the effect of altitude, and vice versa. In addition to that, the association between altitude and CV risk is seen at ≥2500 m.a.s.l. in urban settings and ≥3500 m.a.s.l. in rural ones, highlighting that altitude may be the main factor associated with lower CV risk.

### Implications of the findings

While 50% of the world’s population lives below 150 m.a.s.l, only 6% de the world’s population lives over 1,500 m.a.s.l. [26,27], and most of these people are from low- and middle-income countries (LMIC). Thus, disentangling the potential effect of urbanization and altitude on CV risk seems to be relevant, especially for resource-constrained settings where the burden of CV is high and risk-based prevention much needed. As many of the rural areas are at high altitude, our findings help to better understand the epidemiology of absolute CV risk in these populations. Thus, our results highlight that rurality and high altitude (≥2,500 m.a.s.l.) are negatively and independently associated with high CV risk; however, the potential effect of high-altitude on CV risk is seen at lower altitude among urban dwellers.

Contrary to the usual paradigm that altitude and rurality are associated with a low prevalence and incidence of cardiovascular risk factors (i.e., smoking, obesity, hypertension, and type 2 diabetes), current literature suggest that the distribution of these risk factors in rural and high-altitude settings could have changed. Thus, a recent analysis reported that ≥55% of the global rise in BMI in the last three decades, and ≥80% in some low- and middle-income regions, was attributed to increase of BMI in rural areas [28]. This has been also confirmed in Peruvian less urbanized settings [29]. Whereas cross-sectional studies have reported low prevalence of risk factors in rural settings, a longitudinal analysis in Peru reported a higher risk of developing hypertension in rural areas relative to rural-urban migrant or urban groups [30]. In addition to that, a dramatic change in lifestyle, whereby unhealthy lifestyles are more common, has taken place in Peruvian rural and high-altitude populations. Although urbanization is usually associated to increased consumption of fat, sugar and salt, the results of a study comparing diet patterns in urban and rural settings in a high-altitude Peruvian region reported mixed findings with more added salt and oil consumption than urban areas [31]. Despite of this, according to our findings, subjects with CV risk ≥10% are still less common in rural and high-altitude settings in Peru. A potential explanation of these findings may be the duration of the exposure to these cardiometabolic risk factors; in other words, these rural and high-altitude populations may have been exposed to these risk factors not long enough to have experienced the deleterious effects on cardiovascular health.

Beyond traditional risk factors, altitude may also have a direct effect on cardiovascular physiology. Thus, with chronic altitude exposure, there is a decrease in maximum heart rate, with a decrease in heart rate change during exercise [32]. Similarly, altitude exposure is associated with lower levels of systolic blood pressure [33]. In addition, ultraviolet radiation from sunlight, which tend to increase with altitude, may have a substantial impact on CVD risk [34]. This effect may be mediated by vitamin D levels and by lower blood pressure [35]. Thus, there is a need to understand mechanisms behind the association between altitude and CVD risk.

### Public health relevance

For this manuscript, we used one of the six modules of the HEARTS technical package developed by the World Health Organization (WHO) [18]. The module is focused on risk-based CVD management and includes information on a total risk approach to the assessment of CVD, including subregion-specific risk charts [19].

Despite the limitation of risk scores we used, our findings highlight the need to target individuals at higher risk of CVD, especially those located in urban areas and living <2500 m.a.s.l., but without excluding those from rural or high-altitude settings. Targeting individuals at high CV risk has been recognized as a beneficial strategy in terms of number of CV events avoided [36,37]. Providing CVD risk score may reduce total cholesterol, systolic blood pressure, and multivariable CVD risk [37]. We decided to use an absolute CV risk approach as a subject with multiple risk factors, even if each factor is only slightly or moderately elevated, may be at a higher risk of CVD than someone with an elevated level of a single risk factor.

Finally, whereas a risk stratification approach, as the used by the WHO HEARTS risk score, is mainly suitable to settings with limited resources, our results suggest the need of ensuring laboratory infrastructure to confirm results and conduct follow-up of individuals, appropriate drug therapy availability for primary prevention of CVD, and counseling strategies in different settings, especially in rural areas.

### Strength and limitations

This study benefits from pooled data derived from consecutive nationally representative surveys in Peru. Our study expands to disentangle relationships between urbanization, altitude and predicted CV risk. However, our analysis has some limitations that merit discussion. First, the cross-sectional nature of the data only permits to evaluate association and not causality. Second, across the years, the sampling frame of the Peruvian DHS has changed; thus, the sampling frame of the last three years was not similar to that of the previous years. However, we have used the year-specific sampling weights, stratum, and sampling units. Because of the sampling frame, although there is a possibility to have some bias because of sampling, this is relatively negligible. Moreover, although there is a small probability to select the same participant in different surveys (for example in 2014 and 2020), this probability is very reduced as different sampling frames are utilized. Third, as blood samples are not usually taken in these surveys (i.e., lipid profile and fasting glucose are not available), we only utilized the non-laboratory chart of the WHO absolute CV risk score. However, a moderate agreement between WHO CVD risk predictions using laboratory and non-laboratory algorithms has been described in population samples [18]. Finally, some residual confounding may arise as an exhaustive list of confounders was not used because of data availability.

## Conclusions

Among Peruvian population, urbanization, specifically rurality, and altitude above the sea level, mainly living at ≥2,500 m.a.s.l., were factors independently associated with lower predicted CV risk.

## Data Accessibility statement

Data used in this analysis is freely available in the webpage of the National Institute of Statistics and Informatics (INEI) at http://iinei.inei.gob.pe/microdatos/.

## Additional File

The additional file for this article can be found as follows:

10.5334/gh.1130.s1 Online Supplement. E-Table 1.

## References

[1] Jagannathan R, Patel SA, Ali MK, Narayan KMV. Global Updates on Cardiovascular Disease Mortality Trends and Attribution of Traditional Risk Factors. Curr Diab Rep. 2019; 19(7): 44. DOI: 10.1007/s11892-019-1161-2 31222515

[2] World Health Organization. World Health Statistics 2021: Monitoring health for the sustainable development goals. Geneva, Switzerland: WHO; 2021.

[3] Roth GA, Mensah GA, Johnson CO, et al. Global Burden of Cardiovascular Diseases and Risk Factors, 1990-2019: Update From the GBD 2019 Study. J Am Coll Cardiol. 2020; 76(25): 2982–3021. DOI: 10.1016/j.jacc.2020.11.010 33309175 PMC7755038

[4] Eckert S, Kohler S. Urbanization and health in developing countries: A systematic review. World Health Popul. 2014; 15(1): 7–20. DOI: 10.12927/whp.2014.23722 24702762

[5] Mallet RT, Burtscher J, Richalet JP, Millet GP, Burtscher M. Impact of High Altitude on Cardiovascular Health: Current Perspectives. Vasc Health Risk Manag. 2021; 17: 317–35. DOI: 10.2147/VHRM.S294121 34135590 PMC8197622

[6] Bernabé-Ortiz A, Carrillo-Larco RM, Gilman RH, Checkley W, Smeeth L, Miranda JJ. Impact of urbanisation and altitude on the incidence of, and risk factors for, hypertension. Heart. 2017; 103(11): 827–33. DOI: 10.1136/heartjnl-2016-310347 28115473 PMC5529980

[7] Pajuelo-Ramírez J, Torres-Aparcana H, Agüero-Zamora R, Quispe AM. Altitude and its inverse association with abdominal obesity in an Andean country: A cross-sectional study. F1000Res. 2019; 8: 1738. DOI: 10.12688/f1000research.20707.2 31824671 PMC6896245

[8] Bernabé-Ortiz A, Carrillo-Larco RM, Gilman RH, et al. Geographical variation in the progression of type 2 diabetes in Peru: The CRONICAS Cohort Study. Diabetes Res Clin Pract. 2016; 121: 135–45. DOI: 10.1016/j.diabres.2016.09.007 27710820 PMC5154928

[9] Lazo-Porras M, Bernabe-Ortiz A, Quispe R, et al. Urbanization, mainly rurality, but not altitude is associated with dyslipidemia profiles. J Clin Lipidol. 2017; 11(5): 1212–22.e4. DOI: 10.1016/j.jacl.2017.06.016 28780399 PMC5624786

[10] Ezzati M, Horwitz ME, Thomas DS, et al. Altitude, life expectancy and mortality from ischaemic heart disease, stroke, COPD and cancers: National population-based analysis of US counties. J Epidemiol Community Health. 2012; 66(7): e17. DOI: 10.1136/jech.2010.112938 21406589

[11] Burtscher J, Millet GP, Burtscher M. Does living at moderate altitudes in Austria affect mortality rates of various causes? An ecological study. BMJ Open. 2021; 11(6): e048520. DOI: 10.1136/bmjopen-2020-048520 PMC8183194 34083346

[12] Hernández-Vásquez A, Vargas-Fernández R, Chacón-Diaz M. Association between Altitude and the Framingham Risk Score: A Cross-Sectional Study in the Peruvian Adult Population. Int J Environ Res Public Health. 2022; 19(7). DOI: 10.3390/ijerph19073838 PMC8998056 35409522

[13] Mills KT, Bundy JD, Kelly TN, et al. Global Disparities of Hypertension Prevalence and Control: A Systematic Analysis of Population-Based Studies From 90 Countries. Circulation. 2016; 134(6): 441–50. DOI: 10.1161/CIRCULATIONAHA.115.018912 27502908 PMC4979614

[14] Ebrahim S, Pearce N, Smeeth L, Casas JP, Jaffar S, Piot P. Tackling non-communicable diseases in low- and middle-income countries: Is the evidence from high-income countries all we need? PLoS Med. 2013; 10(1): e1001377. DOI: 10.1371/journal.pmed.1001377 23382655 PMC3558465

[15] Murray CJ, Vos T, Lozano R, et al. Disability-adjusted life years (DALYs) for 291 diseases and injuries in 21 regions, 1990-2010: A systematic analysis for the Global Burden of Disease Study 2010. Lancet. 2012; 380(9859): 2197–223. DOI: 10.1016/S0140-6736(12)61689-4 23245608

[16] Instituto Nacional de Estadística e Informática. Perú: Encuesta Demográfica y de Salud Familiar ENDES 2019. Lima, Perú: INEI; 2020.

[17] Instituto Nacional de Estadística e Informática. Encuesta Demográfica y de Salud Familiar: Ficha Técnica. Lima, Peru: INEI; 2020.

[18] World Health Organization. HEARTS technical package for cardiovascular disease management in primary health care: Risk based CVD management. Geneva, Switzerland: WHO; 2020.

[19] World Health Organization (WHO) CVD Risk Chart Working Group. World Health Organization cardiovascular disease risk charts: Revised models to estimate risk in 21 global regions. Lancet Glob Health. 2019; 7(10): e1332–e45. 31488387 10.1016/S2214-109X(19)30318-3 PMC7025029

[20] Instituto Nacional de Estadística e Informática. Manual de la Entrevistadora: Encuesta Demográfica y de Salud Familiar. Lima, Perú: INEI; 2020.

[21] Rutstein SO, Johnson K. The DHS wealth index. DHS comparative reports No 6. Calverton, Maryland: ORC Macro; 2004.

[22] Chobanian AV, Bakris GL, Black HR, et al. The Seventh Report of the Joint National Committee on Prevention, Detection, Evaluation, and Treatment of High Blood Pressure: The JNC 7 report. Jama. 2003; 289(19): 2560–72. DOI: 10.1001/jama.289.19.2560 12748199

[23] Instituto Nacional de Estadística e Informática. Manual de la Antropometrista: Encuesta Demográfica y de Salud Familiar. Lima, Perú: INEI; 2020.

[24] West BT, Berglund P, Heeringa SG. A closer examination of subpopulation analysis of complex–sample survey data. Stata J. 2008; 8(4): 520–31. DOI: 10.1177/1536867X0800800404

[25] Rao JNK, Scott AJ. On Chi-Squared Tests for Multiway Contingency Tables with Cell Proportions Estimated from Survey Data. Ann Statist. 1984; 12(1): 46–60. DOI: 10.1214/aos/1176346391

[26] Cohen JE, Small C. Hypsographic demography: the distribution of human population by altitude. Proc Natl Acad Sci USA. 1998; 95(24): 14009–14. DOI: 10.1073/pnas.95.24.14009 9826643 PMC24316

[27] Urban Demographics. World population distribution by altitude; 2017 (updated 29 June 2017) https://urbandemographics.blogspot.com/2017/06/world-population-distribution-by.html. (Accessed 22 February 2022).

[28] NCD Risk Factor Collaboration (NCD-RisC). Rising rural body-mass index is the main driver of the global obesity epidemic in adults. Nature. 2019; 569(7755): 260–4. DOI: 10.1038/s41586-019-1171-x 31068725 PMC6784868

[29] Carrillo-Larco RM, Miranda JJ, Gilman RH, Checkley W, Smeeth L, Bernabé-Ortiz A. Trajectories of body mass index and waist circumference in four Peruvian settings at different level of urbanisation: The CRONICAS Cohort Study. J Epidemiol Community Health. 2018; 72(5): 397–403. DOI: 10.1136/jech-2017-209795 29472520 PMC5909748

[30] Bernabe-Ortiz A, Sanchez JF, Carrillo-Larco RM, et al. Rural-to-urban migration and risk of hypertension: Longitudinal results of the PERU MIGRANT study. J Hum Hypertens. 2017; 31(1): 22–8. DOI: 10.1038/jhh.2015.124 26865219 PMC4981561

[31] McCloskey ML, Tarazona-Meza CE, Jones-Smith JC, et al. Disparities in dietary intake and physical activity patterns across the urbanization divide in the Peruvian Andes. Int J Behav Nutr Phys Act. 2017; 14(1): 90. DOI: 10.1186/s12966-017-0545-4 28693514 PMC5504673

[32] Boushel R, Calbet JA, Rådegran G, Sondergaard H, Wagner PD, Saltin B. Parasympathetic neural activity accounts for the lowering of exercise heart rate at high altitude. Circulation. 2001; 104(15): 1785–91. DOI: 10.1161/hc4001.097040 11591615

[33] Riley CJ, Gavin M. Physiological Changes to the Cardiovascular System at High Altitude and Its Effects on Cardiovascular Disease. High Alt Med Biol. 2017; 18(2): 102–13. DOI: 10.1089/ham.2016.0112 28294639

[34] Faeh D, Gutzwiller F, Bopp M. Lower mortality from coronary heart disease and stroke at higher altitudes in Switzerland. Circulation. 2009; 120(6): 495–501. DOI: 10.1161/CIRCULATIONAHA.108.819250 19635973

[35] Krause R, Bühring M, Hopfenmüller W, Holick MF, Sharma AM. Ultraviolet B and blood pressure. Lancet. 1998; 352(9129): 709–10. DOI: 10.1016/S0140-6736(05)60827-6 9728997

[36] World Health Organization. Integrated management of cardiovascular risk: Report of a WHO meeting. Geneva, Switzerland: WHO; 2002.

[37] Karmali KN, Persell SD, Perel P, Lloyd-Jones DM, Berendsen MA, Huffman MD. Risk scoring for the primary prevention of cardiovascular disease. Cochrane Database Syst Rev. 2017; 3(3): Cd006887. DOI: 10.1002/14651858.CD006887.pub4 28290160 PMC6464686

