# Supplementary material for: Urbanization, Altitude and Cardiovascular Risk
Source: Glob Heart. 2022 Jun 21;17(1):42. doi: 10.5334/gh.1130 (PMC9231580; doi:10.5334/gh.1130)
Supplement: Online Supplement. — E-Table 1. [file gh-17-1-1130-s1.pdf]

ONLINE SUPPLEMENT

E-Table 1: Study characteristics comparison between those included and not included in the analyses.

	Not included (n = 5,797)	Included (n = 80,409)	p-value
**Age**			< 0.001
40 – 49 years	2,797 (45.2%)	33,631 (37.8%)	
50 – 59 years	1,553 (26.0%)	23,430 (30.3%)	
60 – 69 years	1,093 (21.1%)	17,191 (23.4%)	
70+ years	354 (7.7%)	6,157 (8.5%)	
**Sex**			0.04
Male	2,659 (43.3%)	37,769 (45.6%)	
Female	3,138 (56.7%)	42,640 (54.4%)	
**Education**			< 0.001
< 7 years	2,044 (28.5%)	31,320 (35.2%)	
7 – 11 years	1,973 (35.8%)	25,234 (35.7%)	
12+ years	1,543 (35.7%)	18,098 (29.1%)	
**Wealth index**			< 0.001
First quintile	232 (2.7%)	17,817 (15.7%)	
Second quintile	1,657 (16.7%)	15,917 (14.2%)	
Third quintile	2,052 (32.7%)	13,551 (16.6%)	
Fourth quintile	1,465 (37.2%)	15,586 (24.7%)	
Fifth quintile	391 (10.7%)	17,538 (28.8%)	
**Current smoking**			< 0.001
No	4,586 (88.4%)	67,755 (85.4%)	
Yes	583 (11.6%)	12,654 (14.6%)	
**Alcohol use**			< 0.001
No	4,356 (82.2%)	55,592 (67.6%)	
Yes	832 (17.8%)	24,768 (32.4%)	
**Hypertension**			< 0.001
No	447 (31.6%)	57,773 (68.4%)	
Yes	888 (68.4%)	22,636 (31.6%)	
**Survey year**			< 0.001
2014	229 (3.6%)	11,910 (12.5%)	
2015	186 (4.0%)	11,426 (15.0%)	
2016	222 (4.7%)	11,478 (14.5%)	
2017	165 (4.0%)	11,877 (15.4%)	
2018	207 (4.6%)	12,545 (16.2%)	
2019	253 (5.8%)	12,395 (15.8%)	
2020	4,535 (73.3%)	8,778 (10.6%)	
